# Using Bacterial Transcriptomics to Investigate Targets of Host-Bacterial Interactions in *Caenorhabditis elegans*

**DOI:** 10.1038/s41598-019-41452-2

**Published:** 2019-04-03

**Authors:** Jason P. Chan, Justin R. Wright, Hoi Tong Wong, Anastasia Ardasheva, Jamey Brumbaugh, Christopher McLimans, Regina Lamendella

**Affiliations:** Department of Biology, Juniata College, Huntingdon, PA USA

## Abstract

The interactions between a host and its resident microbes form complicated networks that can affect host physiology. Disentangling these host-microbe interactions can help us better understand mechanisms by which bacteria affect hosts, while also defining the integral commensal protection that host-associated microbiota offer to promote health. Here we utilize a tractable genetic model organism, *Caenorhabditis elegans*, to study the effects of host environments on bacterial gene expression and metabolic pathways. First, we compared the transcriptomic profiles of *E*. *coli* OP50 *in vitro* (on agar plates) versus *in vivo* (fed to *C*. *elegans* host). Our data revealed that 110 biosynthetic genes were enriched in host-associated *E*. *coli*. Several of these expressed genes code for the precursors and products needed for the synthesis of lipopolysaccharides (LPS), which are important for innate immune and stress responses, as well as pathogenicity. Secondly, we compared the transcriptomic profiles of *E*. *coli* fed to hosts with different genetic backgrounds, including the long-lived *daf-2*/insulin like growth factor (IGF) receptor and short lived *daf-16*/FOXO transcription factor mutants. We find that hosts genetics also alters bacterial metabolic pathways. Given that bacteria influence host health, this transcriptomics approach can elucidate genes mediating host aging.

## Introduction

The interaction between an animal host and its intestinal gut bacteria can have large influences on health and disease. Understanding the activity of the gut microbiota - the microbiome and its metabolites - will help elucidate how bacteria affect host physiology. However, for animals such as humans, the gut microbial community can be comprised of 100 trillion bacteria cells and approximately 400 species of bacteria^1,2^, making it challenging to study the effects of interspecies interactions between the vast gut microbiota and its host. Nonetheless, a healthy gut microbiota is known to influence host immune function, development, metabolism, and aging, whereas a dysbiotic gut microbiome may lead to gastrointestinal tract disorders, diabetes mellitus, and obesity^3^.

Bacteria have coevolved with their animal hosts such that they maintain a symbiotic and necessary interrelationship^4^. In fact, bacteria produce essential and modulatory metabolites that affect host physiology, including immune responses, development^5^, nutrients and lipid metabolism^6^, and lifespan in a variety of organisms^7^. For example, bacteria produce short chain fatty acids that alter signaling in the host immune system. Moreover, bacterial metabolism alters the generation of neurotransmitters such as nitric oxide (NO), serotonin and GABA that have varied effects on host bacterial resistance and neuronal functions^8^. Likewise, host genetics can influence the gut microbiota. For example, studies on the murine leptin gene showed that obese *ob/ob* mice had more Firmicutes but less Bacteriodetes bacteria than *ob/*+ and +/+ littermates, suggesting that genotype alone can alter gut bacterial composition^4,9^. However, less work has been done examining how bacterial gene expression and metabolic networks change in an host environment. Thus, a greater understanding of the gene expression changes that occur in bacteria may help elucidate metabolites that play roles in health and disease.

Technological advances have allowed us to better understand the functions of bacteria within hosts. Specifically, the application of novel, high-throughput sequencing technologies toward bacterial expression profiling have revealed major shifts in the expression of metabolic pathways related to disease pathogenesis in human microbiome^10,11^. Advances in cDNA synthesis and library preparation for high-throughput sequencing have facilitated bacterial expression profiling. In particular, scientists can develop libraries from small quantities (nanograms) of input RNA. In addition, the development of automated pipelines for functional analysis of high-throughput sequence data have enabled the robust and timely analysis of these large -omics datasets. For example, the Biobakery suite of tools enables the user to rapidly quality filter, perform rRNA removal, normalization, and annotation within hours of data generation^12^. These recent breakthroughs in sample preparation and analysis are beginning to afford biologists with the power to investigate the activity of microbial constituents within host environments.

While bacterial transcriptomics studies hold promise in revealing new ways to treat disease and support human health, these approaches have revealed that the expression profiles of human gut microbiomes are subject to wide intra and inter-subject variability^13–16^. Thus, studying bacterial expression systematically in model organisms, such as *Caenorhabditis elegans*, offers much promise for more controlled, hypothesis-driven investigations that disentangle the homeostatic interactions between the host and its resident microbial consortia. Indeed, *C*. *elegans* studies have shown that gut bacteria can shape host lifespan, metabolism of drugs, and immune responses^17–19^. The power of *C*. *elegans* as a model to study host-bacterial interactions is due to worms growing and feeding on monoxenic cultures of bacteria, to the ability to quickly collect large samples, and to track the genetics of worms and the bacteria they consume. Thus, the use of *C*. *elegans* offers a great opportunity to control the host and bacterial genetics and limit variability in mammalian genomics studies. Here we present a new method for investigating gene expression of *Escherichia coli* strain OP50 bacteria within host *C*. *elegans*. Comparisons of bacterial metabolic pathways in cultured, *in vitro* growth conditions versus *in vivo* animal hosts conditions revealed differences in lipopolysaccharide (LPS) and chemotaxis pathways. Furthermore, comparison of bacteria in hosts of wildtype and mutant models of aging show that our bioinformatics platform can to identify bacterial metabolic pathways that may impact host physiology. This proof-of-concept study highlights the bacterial activities of residential *E*. *coli* in the worm gut, revealing potential regulatory mechanisms of these bacterial in on host physiology.

## Results

### Comprehensive transcriptome profile comparisons

**Figure 1:**
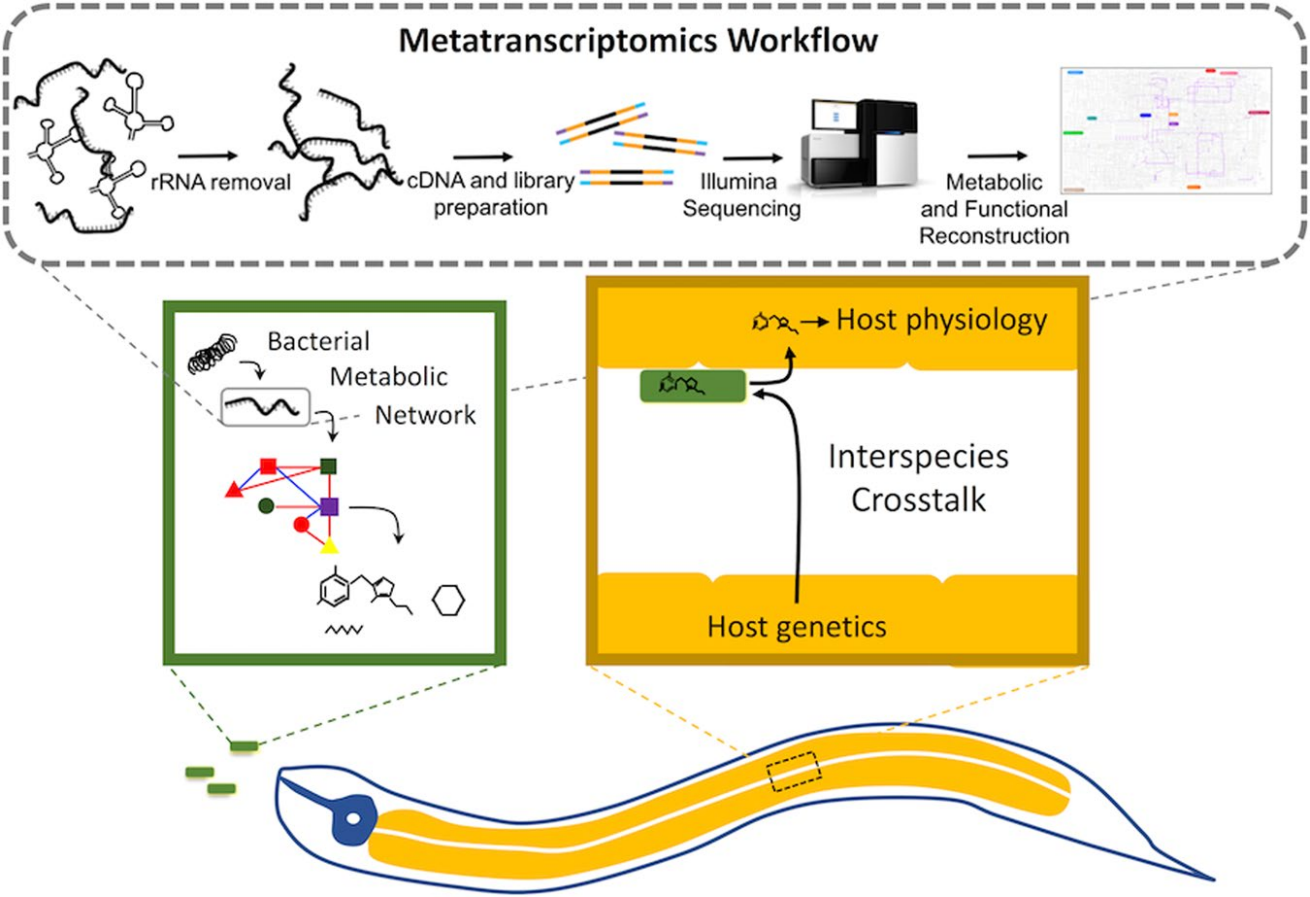
Diagram depicting the crosstalk between bacteria and the *C*. *elegans* host. *Top*, an overview of the transcriptome methods used to profile *E*. *coli* expression in *C*. *elegans*^73–75^. *Bottom*, interspecies interactions, where bacteria have unique metabolic networks that produce metabolites inside the gut of *C*. *elegans*. Host genetics can crosstalk with bacteria and influence bacterial gene expression to affect host physiology.

Given the roles of gut bacteria in myriad aspects of host physiology and disease, we aimed to better understand bacterial gene expression in various environments. For this, we performed transcriptomic analyses to compare gene expression of *E*. *coli* OP50 in two parameters: 1) *in vitro* (on agar plates) versus *in vitro* (in animals hosts) and 2) in varying host genetics that are models of aging. For hosts, we used the *C*. *elegans* model because they survive on one bacterial food source, have tractable genetics, and their physiology and longevity are affected by bacterial metabolites and pathogenesis. To examine bacteria within *C*. *elegans* hosts, we collected worms maintained on *E*. *coli*, extracted and amplified their cDNA to investigate expression profiles with regards to host environment and genotype. A summary of the transcriptome study design is presented in Fig. 1, and sequence quality statistics are provided in Table 1. Validation of transcriptome data is described in the Supplementary Information.

**Table 1:** RNA sequencing results of all experimental sample groups.

Group	Total Raw Reads	Total Reads Post Trimming	C. elegans Mapped and Removed Reads	rRNA Mapped and Removed Reads	Human Mapped and Removed Reads	Reads Mapped to *E*. *Coli* KEGG Genes	Total Identified *E*. *coli* KEGG Genes
*in vitro*	88,606,975	64,900,392	180,469	598,393	3,275,756	48,010,748	2,693
*in vitro* + FUdR	60,603,341	42,219,206	103,657	797,716	4,608,927	25,237,929	2,743
WT-15	50,868,500	27,291,467	16,213,328	1,781,955	1,021,279	106,507	2,196
WT-20	52,174,940	32,831,142	9,428,396	2,490,185	8,954,799	384,427	2,550
*daf-2*	52,253,677	26,745,313	13,558,831	2,545,222	897,672	193,464	2,621
*daf-16*	64,960,284	40,064,923	16,515,900	2,632,628	7,492,726	555,950	2,667

**Figure 2:**
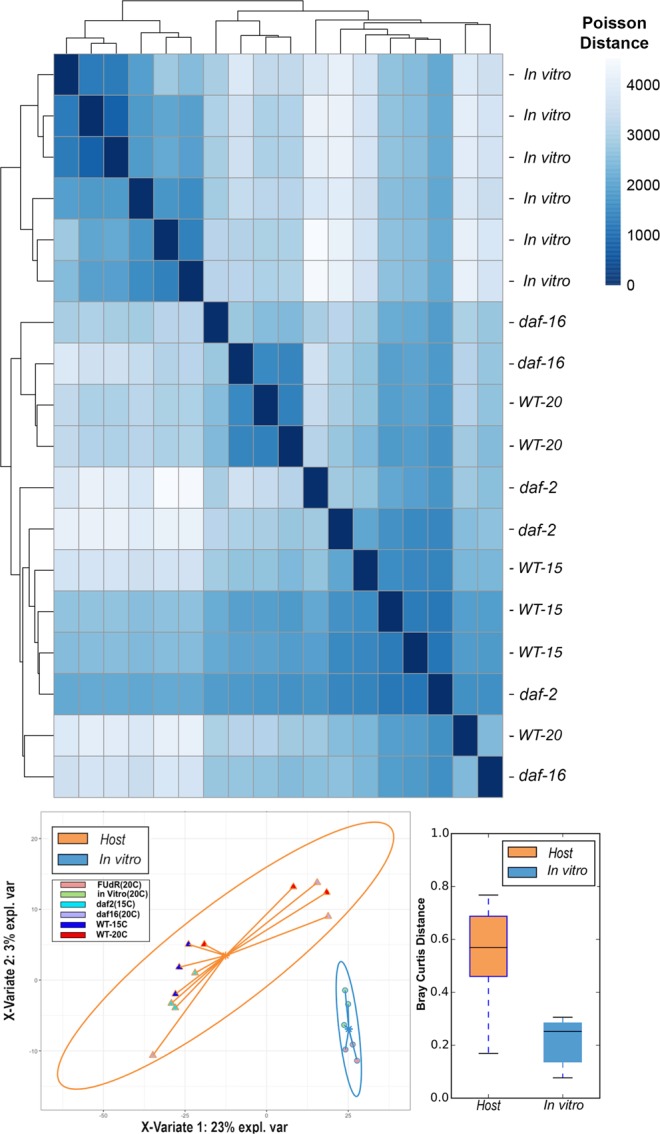
(**A**) Hierarchical Clustering of Bacterial Expression Profiles based on Poisson distance. (**B**) Partial least squares discriminant analysis (PLS-DA) loading plot of *E*. *coli* transcriptomes and (**C**) Bray Curtis distance boxplot comparing variation within Host-associated and *in vitro* sample expression profiles, respectively. (**A**) A hierarchical clustering heatmap displays overall differences in metatranscriptome profiles between collected samples (n = 18). Poisson distances between expression profiles are represented from low to high, indicated by darker to lighter shading, respectively. Samples clustering closely share similar functional gene expression profiles, whereas distant samples yield distinct expression profiles. Poisson distances were calculated from a DEseq. 2 object consisting of CPM normalized uniref50 annotations. Uniform clustering of *in vitro E*. *coli* OP50 transcriptomes can be observed at the top of the heatmap, with worm-associated samples clustered below. (**B**) Partial least squares discriminant analysis (PLS-DA) was conducted within the mixOmics R-package utilizing a CPM normalized KEGG Orthology (KO) counts table of genes mapped to *E*. *coli*. The solid ellipses around sample groups indicate 95% confidence. Here, expression profiles generated from *C*. *elegans* Host-associated *E*. *coli* are highlighted in orange, whereas cultivated *E*. *coli* expression profiles (*in vitro*) are highlighted in blue. Components with most explained variance were chosen for PLS-DA clustering. A lack of overlap between cohorts indicates a defined gene expression profile within the respective cohorts. (**C**) Increased variance in expression profiles were observed between Host-associated samples in comparison to *in vitro* samples. Greater distances in the *E*. *coli* expression profiles were observed within worm-associated samples (average Bray Curtis distance = 0.56) in comparison to *in vitro* samples (average Bray Curtis distance = 0.21).

**Figure 3:**
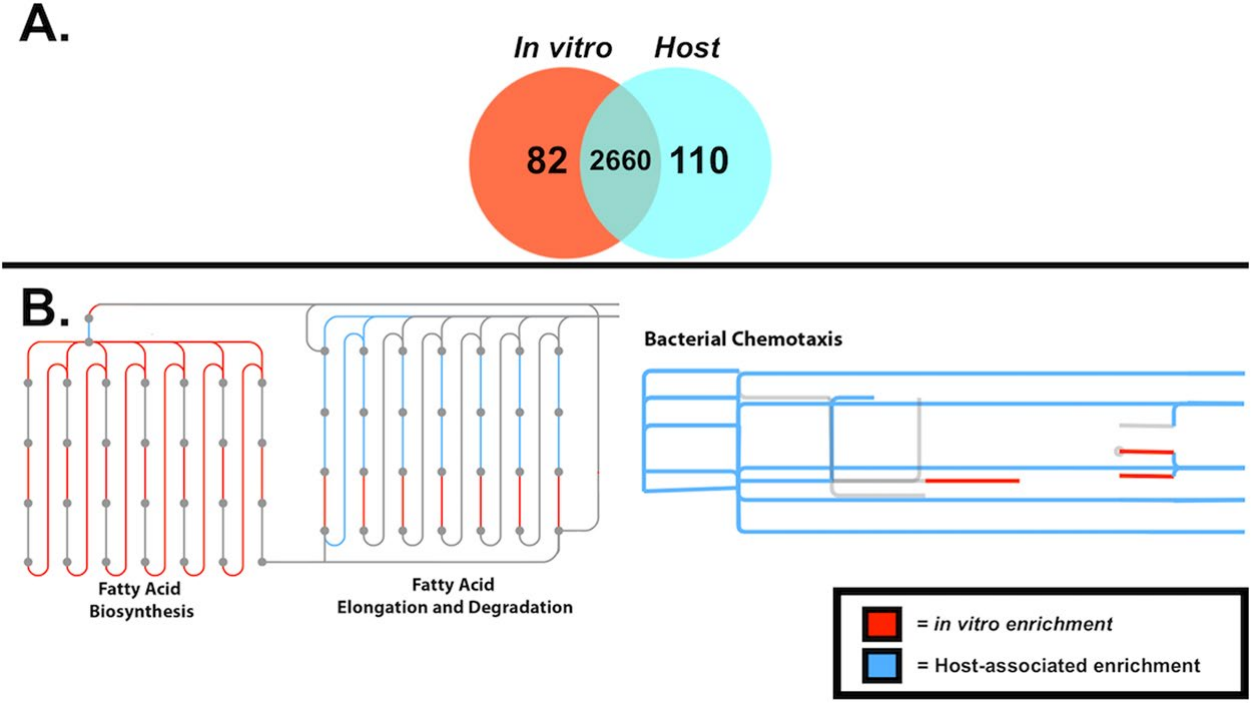
(**A**) Venn Diagram of shared and unique KEGG Orthology terms between *in vitro* (+FUdR) and host-associated *E*. *coli*. Counts of KEGG orthologies (KOs) identified within at least one host-associated *E*. *coli* or *in vitro* sample exclusively were quantified to compare shared and unique functional gene counts between the two sample groups. A total of 110 genes were expressed exclusively within host-associated samples, whereas 83 were exclusive to *in vitro* samples. Over 2500 shared genes were expressed by at least one sample within each group. (**B**) Significantly differential KEGG orthologies identified between pairwise comparisons of host-associated and *in vitro* samples mapped to iPath bacterial chemotaxis and fatty acid biosynthesis, elongation and degradation pathways. iPath plots provide a comprehensive overview of the summarized function of significantly differential functional genes between *in vitro* and host-associated samples. Here, significantly differential KEGG orthologies were mapped onto the KEGG reference *Metabolic Pathway* map. Regions of the reference pathway highlighted in red are upregulated within *in vitro* samples, whereas pathways highlighted in blue are increased in expression within host-associated samples. Grey regions are not significantly differential between the two conditions. *E*. *coli* upregulation (LDA > 0.5, P < 0.05) of genes related to fatty acid biosynthesis were observed within *in vitro* samples in comparison to host-associated samples, whereas fatty acid elongation and degradation genes were identified to be overexpressed within host-associated samples. Conserved enrichment of bacterial chemotaxis genes were observed within host-associated samples when compared to *in vitro* counterparts.

*E*. *coli* gene annotations within the Kyoto Encyclopedia of Genes and Genomes (KEGG) database yielded distinct clustering of *in vitro* samples from host-associated *in vivo* samples (*WT*, *daf-16*/FOXO, *daf-2*/IGF-1 receptor) (Fig. 2). Regarding within-host comparisons, a majority of the wild type host-associated *E*. *coli* transcriptomes (WT) yielded distinct profiles from the mutant worm-associated profiles (*daf-16*/*daf-2*). Reads per kilobase per million sequence (RPKM) counts of KEGG Orthologies (KOs) were incorporated into a partial least squares discriminant analysis (PLS-DA) model, and revealed distinct clustering of samples between *in vitro* and host-associated samples (Fig. 3A). Principal coordinates analysis (PCoA) revealed that overall transcriptome expression profiles of *E*. *coli* from host-associated and *in vitro* samples were significantly different considering the Bray Curtis distance metric (Adonis PERMANOVA P < 0.05). In addition, elevated variation in expression profiles was observed within host-associated *E*. *coli* samples (average distance = 0.56) in comparison to *in vitro E*. *coli* (average distance = 0.21) (Fig. 3B).

### Host-associated vs. *in vitro* functional gene comparisons

Given that bacteria *in vitro* face different conditions than bacteria in hosts, we aimed to determine how functional gene expression of *E*. *coli* change in the two environments. Differential *E*. *coli* gene expression analysis revealed a higher count of upregulated genes under *in vitro* conditions (357 genes), as compared to *E*. *coli* sampled within wild type (*WT*) worms (254 genes) as summarized in Supplementary Table 1. The biofilm regulator *BssS* was identified as the most significantly enriched KO within the *in vitro* cohort (LDA = 4.26, P < 0.05). Within the WT cohort, upregulation of several genes involved in lipopolysaccharide (LPS) biosynthesis including LPS biosynthesis gene *wzzE* (LDA = 2.00, P < 0.05) and glucosyl LPS alpha-1,3-glucosyltransferase (*waaO*) (LDA = 1.70, P < 0.05) were found to be overexpressed.

*C*. *elegans* consume bacteria as a nutrient source, but bacteria can accumulate within host intestines and impact host physiology. In particular, bacteria within *C*. *elegans* have been known to contribute to host aging and immunity^20^. Thus, we aimed to determine whether gene expression profiles differed in bacteria accumulating in mutant hosts, specifically those with aging phenotypes, compared to bacteria *in vitro*. First, we analyzed bacteria in animals with mutations in the insulin/IGF receptor ortholog *daf-2*, which have longer lifespans than wildtype animals^21^. When considering *E*. *coli* gene expression comparisons between *in vitro E*. *coli* and 4-day old *daf-2* mutants, 851 significantly differential expressed genes were observed. A total of 320 and 531 KOs were overexpressed within the *in vitro* and *daf-2* groups, respectively (Supplementary Table 2). Genes involved in lipopolysaccharide biosynthesis (LPS) and the two-component ompR gene family were enriched within *daf-2* associated *E*. *coli*. Three genes involved in LPS biosynthesis including LPS biosynthesis gene *wzzE*, were enriched within the *daf-2* associated *E*. *coli* (LDA = 2.02, P < 0.05). The LPS export system permease gene (*lptG*, LDA = 1.69, P < 0.05) and glucosyl LPS alpha-1,2-glucosyl/galactosyltransferase (*waaR*, LDA = 2.15, P < 0.05) were also found to be upregulated within *daf-2* worms. Two-component genes within the ompR gene family including phosphate regulon response regulator *phoB*, KDP operon response regulator *kdpE*, catabolic regulation response regulator *creB*, sensor histidine kinases *kdpD* and *torS* were amongst 7 significantly enriched ompR cassette genes within *daf-2* associated *E*. *coli*. Aerobic respiration control protein a*rcA* and response regulator *phoP* were the only ompR genes identified as enriched within *in vitro* samples in comparison to *daf-2* associated *E*. *coli*.

Next, we analyzed bacteria in animals with mutations in the FOXO transcription factor *daf-16*. DAF-16 is a downstream target of DAF-2/IGF receptor, and thus have short lifespans^22^. A total of 439 significantly differential expressed functional genes were identified between *in vitro* and 4-day old *daf-16* animals; 130 were identified as enriched within the *in vitro* samples, and 319 within the *daf-16* cohort (Supplementary Table 3). Interestingly, 17 of the overexpressed genes identified within the *daf-16* cohort mapped to two-component system pathways, of which 7 belong to the ompR family, including: *creC*, *cusS*, *torS*, *arcB*, *rstA*, *kdpE*, *and basR* (LDA > 1.0, P < 0.05, Supplementary Table 3). A single two-component pathway gene, *cpxR*, also within the OmpR family, was identified as overexpressed within the *in vitro* samples. The *CheA* (LDA = 1.69, P < 0.05) sensor kinase, *CheB* (LDA = 1.36, P < 0.05) response regulator, and *CheZ* (LDA = 1.44, P < 0.05) chemotaxis family two-component system genes were all overexpressed within the *daf-16* cohort as compared to their respective *in vitro* group. A single LPS biosynthesis gene TDP-4-oxo-6-deoxy-D-glucose transaminase (*wecE*) was also enriched within the *daf-16* cohort (LDA = 1.28, P < 0.05).

In total, 110 unique KOs were observed exclusively within host-associated samples, whereas 82 were identified solely within *in vitro* samples (Fig. 3A). Genes exclusive to host-associated *E*. *coli* samples mapped to two-component regulatory systems, including the AtoS-AtoC (cPHB biosynthesis) two-component regulatory system (atoC) and Ttrs-TtrR (tetrathionate respiration) two-component regulatory system (ttrs). Lipopolysaccharide biosynthesis protein *waaB* and beta-Lactam resistance gene *blaZ* were also observed to be exclusive host-associated *E*. *coli* samples (Supplementary Table 4). Genes exclusive to *in vitro* samples included methionine salvage pathway genes *mtnB*, *mtnD* and *mtnE* (Supplementary Table 5).

To better visualize functional, comprehensive pairwise differences between *in vitro* and host-associated *E*. *coli* transcriptomes, significantly differential expressed genes were mapped to comprehensive metabolic KEGG pathways (Fig. 3B). A total of 1,679 significantly differential genes were successfully mapped to functional gene pathways within iPath 3. *E*. *coli* from hosts showed enriched expression of genes related to bacterial chemotaxis and fatty acid degradation and elongation pathways compared to *E. coli* grown *in vitro*. Conversely, *E*. *coli* grown *in vitro* exhibited enrichment of genes mapped to the fatty acid biosynthesis pathway compare bacteria grown in animal hosts. An iPath formatted table for interactive data mapping is provided in Supplementary Data 1.

We further identified genes expressed in all *in vitro and in vivo E*. *coli*, regardless of environment, as these genes most likely represent core genes found in *E*. *coli* bacteria. This analysis revealed 192 conserved KEGG orthologies across all 18 samples (Supplementary Table 6). Of these core genes 65 were successfully mapped to the iPath 3 Metabolic Pathway map, and revealed a conservation of genes involved in carbohydrate metabolism including the Pentose Phosphate Pathway (Supplementary Fig. 1). Transketolase A/B (tktA,tktB), transaldolase A/B (talA,talB) and 6-phosphofructokinase 1 (pfkA) were all identified as core genes across all samples that fall within the Pentose Phosphate Pathway. We also identified several tRNA biosynthesis genes required for translational activity within *E*. *coli* including tryptophanyl-tRNA synthetase (WARS), threonyl-tRNA synthetase (TARS), leucyl-tRNA synthetase (LARS), alanyl-tRNA synthetase (AARS) and glutaminyl-tRNA synthetase were all identified as core genes within the dataset. Core genes related to glycolysis including 6-phosphofructokinase 1 (pfkA), pyruvate kinase (PK) and phosphoglycerate kinase (PGK) were also common across transcriptomes. Interestingly, drug resistance genes including a multidrug efflux pump (acrB), MFS transporter tetracycline resistance protein (tetA) and beta-lactamase class A were also identified as core genes across the dataset.

### Functional gene expression varies depending on host-genotype

To determine whether host genetic background has an impact on bacterial gene expression, we examined expressed *E*. *coli* functional genes (KOs) within wildtype and mutant *C*. *elegans*. To gain a better understanding of the bacterial metabolic networks that may contribute to the physiology of animal models of aging, we compared the transcriptome of 4-day old wild type and the long-lived *daf-2/*IGF-1 *recepto*r mutants and the short-lived *daf-16/*FOXO mutants. DAF-16/FOXO is a downstream target DAF-2/IGF-1 receptor; activation of the DAF-2/IGF-1 receptor initiates an AGE-1/PI3K signaling cascade that inhibits DAF-16/FOXO from entering the nucleus and driving gene transcription. Genes regulated by DAF-16/FOXO include stress response genes that mediate oxidative stress resistance and animal survival, amongst others. Thus, mutations in *daf-2/*IGF receptor confer longer lifespans, whereas mutations in *daf-16*/FOXO confer short lifespans.

**Figure 4:**
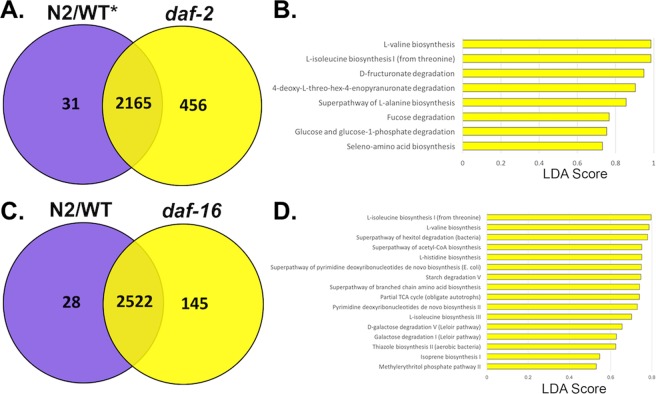
(**A**) Venn Diagram of shared and unique KEGG Orthology terms between WT-15 and *daf-2* host-associated *E*. *coli*. A total of 31 unique WT-15 KEGG orthology genes (KO) and 456 unique *daf-2* KOs were identified. Over 2100 overlapping KOs were shared between both cohorts. **(B**) LEfSe plots display significantly enriched Metacyc functional pathways *within daf-*2 associated *E*. *coli* (p < 0.05, LDA > 0.5). No unique enriched pathways identified in the WT-15. The Y-axis displays the pathway enriched in respective cohort (separated by color) and X-axis displays the LDA score of each pathway, which quantifies the strength of enrichment. **(C**) Venn Diagram of shared and unique KEGG Orthology terms between WT-20 and *daf-16* host-associated *E*. *coli*. A total of 28 unique WT-15 KEGG orthology genes (KO) and 145 unique *daf-2* KOs were identified. Over 2500 overlapping KOs were shared between both cohorts. **(D**) LEfSe plots display significantly enriched Metacyc functional pathways *within daf-*16 associated *E*. *coli* (p < 0.05, LDA > 0.5). No unique enriched pathways identified in the WT-20. The Y-axis displays the pathway enriched in respective cohort (separated by color) and X-axis displays the LDA score of each pathway, which quantifies the strength of enrichment.

A total of 225 significantly differentially (LDA > 1.5, P < 0.05) expressed functional genes (KOs) were identified between *daf-2* (long-lived) and respective wildtype (WT-15) samples (Supplementary Table 7). One-hundred and sixty-seven differential KOs were overexpressed by *daf-2* associated *E*. *coli*, whereas 58 KOs were identified as enriched within the WT cohort. Venn diagrams were generated to quantify shared and unique genes within wildtype and *daf-2* cohorts, and revealed 31 and 456 KOs were unique to WT and *daf-2* samples, respectively (Fig. 4A). A Metacyc functional pathway LEfSe enrichment plot revealed eight significantly enriched (LDA > 0.5, P < 0.05) pathways within the *daf-2* samples, including a several different amino acid biosynthetic pathways (LDA > 0.5, P < 0.05) and fucose degradation (LDA = 0.767, P < 0.05) (Fig. 4B).

**Figure 5:**
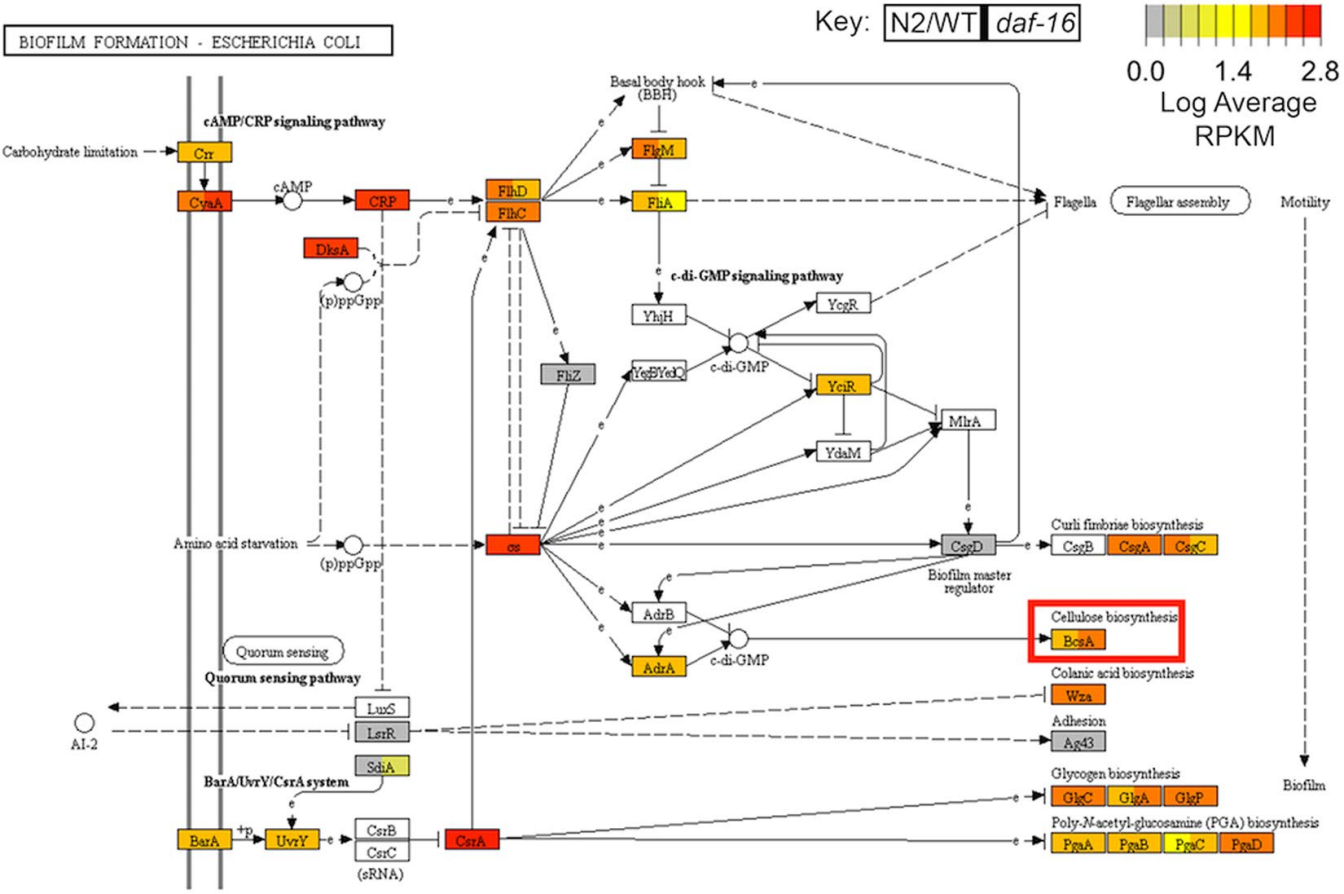
Pathview plots reveal differences in the average expression of functional genes of interest related to biofilm formation between the *daf-16* and WT sample cohorts. CPM normalized counts of KEGG orthologies were mapped to the *Escherichia coli Biofilm Formation* pathway^73–75^ (ko02026) using the R package pathview and revealed elevated expression of genes related to cellulose and glycogen biosynthesis within *daf-16* associated *E*. *coli*. Each rectangle on the plot is indicative of a functional gene within the biofilm formation pathway, and is colored by the respective average expression (log transformed RPKM count) within WT (left) and *daf-16* (right) associated *E*. *coli* with grey indicating no expression, and red indicating high expression. A 3.24 fold increase in the average RPKM normalized count of *BcsA cellulose synthase* was observed within *daf-16* samples in comparison to the WT group as indicated by the red box. Production of biofilm consisting of cellulose in *E*. *coli* has been linked to protection of the bacteria from killing in *C*. *elegans* hosts.

Next, we compared bacterial profiles between *daf-16*/FOXO and wildtype animals. A total of 162 significantly differential (LDA > 1.5, p < 0.05) KEGG orthologies were identified between wildtype (WT-20) and *daf-16* samples (Supplementary Table 8). Within the *daf-16* cohort, 133 overexpressed genes were identified, whereas only 29 KOs were overexpressed in the wildtype transcriptomes. Four KEGG genes annotated as two-component system genes within the *ompR* family including: *basR*, *torS*, *coxA*, and *kdpE* were all enriched within the *daf-16 E*. *coli* transcriptomes. A comparison of shared and unique functional genes within *daf-16* and respective wildtype samples revealed 28 and 145 KOs, unique to wildtype and *daf-16* samples, respectively*;* 2522 KOs shared by both cohorts (Fig. 4C). A total of 15 significantly enriched MetaCyc functional pathways were observed within the *daf-16* cohort, whereas none were identified within the WT *E*. *coli* transcriptomes (Fig. 4D). The starch degradation V MetaCyc pathway amongst the summarized functional observed as significantly enriched (LDA = 0.75, P < 0.05) within the *daf-16* group. Pathview plots revealed differences in the expression of functional genes of interest related to biofilm formation between the *daf-16* and WT sample groups (Fig. 5). Increased expression of the cellulose biosynthesis gene *bcsA* was observed within the *daf-16* sample group. A 3.24-fold increase in the average RPKM normalized count of *BcsA cellulose synthase* was observed within *daf-16* samples in comparison to the WT group. When comparing the expression of the same *BscA cellulose synthase* gene between *daf-2* and respective WT samples, an increased expression was observed within the WT cohort (1.12-fold change).

### Temperature-specific variability in *E*. *coli* gene expression

To assess the variability in the overall transcriptome profile between samples based on incubation temperature, principal coordinate analysis was repeated and revealed bray curtis distances between WT-15 and WT-20 worms (worms cultivated at 15 °C and 20 °C, respectively) to be insignificant (p = 0.331). Follow up LEfSe analysis was conducted to identify temperature-specific biomarker genes within 15° C and 20° C wildtype worms. Identified differential genes (LDA > 2.0, P < 0.05) between WT-15 and WT-20 samples are summarized in Supplementary Table 9. The biofilm regulator protein (*Bsss*) was observed to be significantly overexpressed within WT-20 worms, as well as an LPS assembly lipoprotein (KEGG orthology: K03643).

## Discussion

### Transcriptome profiles of *in vitro* and Host-Associated *E*. *coli* OP50 reveals mechanism of survival

A commensalism exists in the gut between the host and its resident microbes, where microbes experience an anaerobic environment, abundant in nutrients and in return, gut microbes support the host by synthesizing and metabolizing a variety of vitamins and nutrients. Due to recent advances in transcriptomics, for the first time, this study unravels this complex host-microbial interface in the *C*. *elegans* model system. Improvements in massively parallel high-throughput sequencing and in RNA-seq library preparation on low concentration samples have enabled very comprehensive coverage of expressed genes across a large dynamic range of expression (i.e. rare and abundant transcripts)^23^. In addition, transcriptomics approaches provide a more informative perspective on residential gut microbes, as this technology provides information beyond the genetic potential of the organism, generating a snapshot of the bacterial expression under any given condition.

Here we present, for the first time, a transcriptomics approach to measure gene expression of *Escherichia coli* strain OP50 bacteria within host *C*. *elegans*. In addition, our transcriptomics workflow enabled us to evaluate the impact of host genotype on bacterial gene expression. Here we generated a non-redundant list of expressed genes from *E*. *coli* strain OP50 by using transcriptomics profiling of this bacterium isolated from NGM plates only and bacterium isolated from wildtype, *daf-2/*IGF-1 receptor and *daf-16/*FOXO *C*. *elegans* hosts. Most *C*. *elegans* laboratories use the *E*. *coli* strain OP50, yet the interactions between OP50 bacterial gene expression and the host are largely unknown^24,25^. Results of this study demonstrate differential gene expression of between *in vivo* and *in vitro* treatments, with host-associated bacteria overexpressing genes involved in protecting and responding to its dynamically changing environment. Our transcriptomics approach also revealed, for the first time, differences in *E*. *coli* expression based on *C*. *elegans* genotype (*WT*, *daf-2*, and *daf-16*), yielding bacterial functions/targets that could be involved in differentially aging hosts.

### Global transcriptome profiling and differential expression of the *in vivo* and *in vitro E*. *coli* transcriptomes

Large differences were noted between *in vitro* and *in vivo E*. *coli* transcriptomes, which shared less than 47% similarity based on average bray curtis distance calculated between *in vivo* and *in vitro* samples. In addition, a higher variability was observed amongst the different *in vivo* cohorts compared to *in vitro* samples, suggesting that differences in the transcriptomic profiles are not only due to bacteria colonizing the gut, but also to the environmental niche generated by the host. This larger expression variability within *in vivo* versus *in vitro* experiments has been recapitulated by others^26^. A majority of the enriched pathways in the *in vitro* samples mapped to essential components of aerobic and anaerobic respiratory chains, and fatty acid and amino acid biosynthesis. A plethora of biosynthesis, precursor metabolite generation, and energy utilization pathways were overexpressed in cultured *E*. *coli* bacteria, as supported by previous other *in vitro* studies^27,28^.

Interestingly, several *E*. *coli* genes involved in the biosynthesis of precursors and products for bacterial Lipopolysaccharides (LPS) were overexpressed *in vivo* compared to cultured *E*. *coli* OP50 samples. LPS molecules are in the outer bacterial membrane of gram negative bacteria such as *E*. *coli*, providing a protective function for the bacterium especially under stressful environmental conditions experienced within the host^29^. In addition, these molecules are a hallmark of host-microbial interactions, as LPS is an important pathogen associated molecular pattern recognized by host innate immune system. There is some evidence that the *C*. *elegans* nervous system can mediate its interaction with gut bacteria differing in pathogenicity, and the differential neuronal response is mediated by LPS structure^30–32^. The coevolved interaction between residential gut microbes and their host is only beginning to be understood and differential gene expression studies can begin to shed light on the interactive mechanisms by which microbial consortia regulate host physiology. Previous studies have shown evidence for several diverse mechanisms of differential expression related to survival and pathogenicity *in vivo*^33^. This study has shown that *in vivo* gene expression profiles are highly differentiated, providing evidence that the host environment and genotype have a stronger impact on the expression profile than *in vitro* conditions. The high resolution of our transcriptomics profiling experiments enabled us to dissect interactions with the host, and functional alterations that were selected to promote survival and persistence within the host environment.

Several fatty acid synthesis genes were more highly expressed within the *in vitro E*. *coli*, while fatty acid elongation and degradation pathways were more expressive within host-associated *E*. *coli*. An enrichment of endogenous fatty acid biosynthesis genes could be a hallmark of nutrient availability and reduced competition within media conditions. Contrastingly, in host-associated *E*. *coli*, we noted acyl coenzyme A synthetase and other fatty acid metabolism genes were overexpressed, and these genes are required for degradation of long-chain fatty acids, which has recently been shown to decrease pathogenicity of some bacteria^34^. Other hallmarks of lipid degradation by *E*. *coli* in the worm gut included overexpression of ethanolamine lyases. Ethanolamine is derived from the membrane phospholipid phosphatidylethanolamine, and serves as a carbon and/or nitrogen source for bacteria that can catabolize it. The detection of ethanolamine has been proposed as a general mechanism by which bacteria sense their intestinal environments^35^. In addition to being a nutrient source, ethanolamine is a signal for virulence gene expression and current research demonstrates its emerging role in infection and colonization in the intestine. Our study has demonstrated the utility of bacterial transcriptomics to detect the active metabolism of bioactive lipids and their potential role in host–microbial interactions.

Elevated expression of genes within the *E*. *coli* chemotaxis functional pathway were observed in host-associated samples in comparison to *in vitro*. Within *E*. *coli*, sensitive and precise chemotactic responses to fluctuations in the surrounding chemical gradients rely on the expression of membrane bound chemotaxis receptors (MCPs) as well as the *Che* chemotaxis-family gene cassette^36^. All five of the *E*. *coli* methyl-accepting transmembrane chemotaxis receptor proteins including *tsr*, *tar*, *trg*, *tap*, and *aer* were identified to be upregulated within host-associated samples^37^. Conserved elevated expression of *E*. *coli* chemoreceptors within host-associated samples suggest an elevated sensor response to external attractant and repellent stimuli within the *C*. *elegans* host in comparison to pure culture conditions. The enrichment of chemotactic receptors for α-amino acids attractants L-serine and aspartate, *tsr* and *tar* respectively, indicate increased bacterial interactions with host-associated attractants within *in vivo* conditions^38^. We also found that the Aer aerotaxis receptor was elevated within host associated samples. Aer and tsr receptors are thought to sense internal redox states and mediate *E*. *coli* movement towards optimal oxygen conditions^39^ Aer and tsr chemoreceptors in *E*. *coli* mediates movement towards oxidizable sources or those that mediate maximal growth, respectively^40^. Such increased motility is potentially due on the need for aerobic *E*. *coli* to seek and locate oxygen rich conditions within the anaerobic gut of C. *elegans*, and consequently yielding elevated aerotaxis receptor signaling in comparison to aerobic culture conditions^41^.

### Differential expression of *E*. *coli* transcriptomes in models of aging

Host genetics may alter the expression profile of bacteria, which thereby influences bacterial metabolism, growth, and virulence. Previously, it was also found that host genotype can alter the diversity of the *C*. *elegans* host microbiome, but less is known regarding hosts’ effects on bacterial gene expression. Genes that promote bacterial growth and survival in host intestines may lead to bacterial accumulation and impacts on aging. Interestingly, *daf-2/*IGF-1 receptor mutants have less bacterial colonization than age matched wild type animals, suggesting that the abundance of bacteria may be a precursor to age-associated fatality^42^. Interestingly, *daf-2* mutants have been shown to be resistant to infection by *Staphylococcus aureus* and *Pseudomonas aeruginosa*^43,44^. At the same time, bacterial metabolism may affect the production of metabolites that affect host physiology, including aging^45,46^. Our transcriptomic profiling from bacteria found in wildtype and *C*. *elegans* mutants also indicated that *E*. *coli* express different genes in different hosts based on genetic background. Specifically, we identified genes that might promote bacterial adaptation to hosts. One effect was on osmotic regulation genes, in particular the ompR family of the two-component system that senses and adapts to the environment. Two-component system consists of a sensor protein-histidine kinase (HK) and a response regulator (RR). We found that bacteria in short-lived *daf-16* mutants had upregulated *kdpE* and *torS* compared to wildtype; interestingly, this was not observed in long-lived *daf-2* mutants. The *kdpE* system regulates potassium homeostasis and virulence in some bacteria including *E*. *coli*^47^. Two-component genes were also increased in fluoroquinolone-resistant, *Salmonella typhimurium* in a *C*. *elegans* host model^48^.

In addition, we found genes that aided in biofilm formation in bacteria. The expression of the bacterial cellulose pathway gene *bcsA* was higher in short-lived *daf-16/FOXO* hosts compared to wild type hosts. The *bscA* gene produces a cellulose synthase enzyme that is important for biofilm formation in bacteria. Interestingly, production of biofilm in *E*. *coli* protects the bacteria from being killed in *C*. *elegans* hosts^49^. Thus, it is curious to speculate that *E*. *coli* biofilms protect bacteria in *daf-16* mutants, which may contribute to their short lifespan phenotype. Intriguingly, *in vitro* samples exhibited increased *bssS* expression, which has been linked to negatively regulate biofilm formation in *E*. *coli*^50^. Furthermore, loss of biofilm formation in *P*. *aeruginosa* may reduce its virulence effect on worms^51^. Additionally, *S*. *aureus* increases gene transcription of *kdpE*, which was increased with short lived *daf-16* mutants, during biofilm formation. Interestingly, biofilms may also be important for bacteria that promote lifespan extension. For example, biofilm formation by *B*. *subtilis* results in lifespan extension of the *C*. *elegans* host, and this is dependent on *daf-16* and inhibition of the insulin signaling pathway^52,53^; accordingly, worms fed *B*. *subtilis* live longer than those fed *E*. *coli*^54^.

Shifts in the transcriptional profile of host-associated *E*. *coli* were observed between long-lived *daf-2* mutant *C*. *elegans* and respective WT samples (WT-15). In particular, the overexpression of a peroxiredoxin bacterioferritin comigratory protein (peroxiredoxin Bcp) was observed within the *daf-2* mutant associated *E*. *coli*. Peroxiredoxins are a pervasive family of antioxidant enzymes that reduce peroxides (Rhee), hypothesized to possess an anti-aging role, and are ubiquitous across nearly all organisms. The *C*. *elegans* genome contains only a single peroxiredoxin gene (PRDX-2), and thus the suppression of this gene and its linkage to aging has been a topic of interest in previous literature^55^. Such studies have concluded suppression of the *C*. *elegans* PRDX-2 peroxiredoxin is associated with a decreased lifespan of the host^56^. While the anti-aging role of host *C*. *elegans* peroxiredoxin is known, the impact of elevated expression or repression of bacterial peroxiredoxin genes within the host gut microbiome is poorly understood. Within *E*. *coli*, peroxiredoxin Bcp catalyzes the reduction of hydrogen peroxide and other organic hydroperoxides^57^. Peroxiredoxin Bcp possesses the highest redox potential of all known *E*. *coli* peroxiredoxin genes, and thus maintains the ability to reduce both small and large hydro-peroxide substrates even in high oxidizing conditions. The pathogenic bacteria *S*. pyrogenesare known to kill *C*. *elegans* with biosynthesized hydrogen^58^. Therefore, the significantly increased expression of peroxiredoxin Bcp by *E*. *coli* as a stress response mechanism, may be serving as a mutualistic benefit to the host, as it reduces harmful peroxides from within the host *C*. *elegans* gut.

For this study, worms were grown for 4 days (L4 stage + 2 days), which may limit the interpretations of our results. Portal-Celhay *et al*. (2012) found that bacteria, including *Escherichia coli* and *Salmonella typhimurium*, can be observed in wildtype, *daf-2*, *daf-16*, and other *C*. *elegans* mutants to varying quantities at this age^59^. In this study, they also found that bacteria in a wild type worm has less than 10^2^ cfu (colony forming units) at L4 stage, approximately 5 × 10^2^ cfu at 4 days (L4 stage + 2 days) and 10^4^ cfu by day 6 (L4 + 4 days), and remain level through day 10 (L4 + 8 days). Similar results were found using GFP labeled OP50^60^. Thus, we captured bacterial gene expression during growth, and gene expression may change at later ages to impact host physiology. Further studies are needed to specifically address the roles of bacterial metabolism at different growth stages and host ages.

### Future work

The ability to control bacterial and host genetics in the interspecies *C*. *elegans–E*. *coli* interaction has elucidated the role of host environment on changes in bacterial gene expression and metabolic pathways. Bacterial metabolites have a large impact on host stress, immune function, and health. The ability to elucidate bacterial genes and metabolic pathways in specific host genetics or bacteria may identify novel metabolites produced by bacteria that influence host physiology. Interestingly, our study identified genes involved in colanic acid biosynthesis that were upregulated in bacteria of long-lived *daf-2/*IGF-1 receptor mutants, including UDP-glucose 4-epimerase and several wcaF and wcaB genes, which were previously identified in the bacterial screen for genes extending lifespan. Functional studies will be required to confirm the role of the pathways identified here in aging. With an established molecular protocol and bioinformatics pipeline, future work can also address the roles of bacterial metabolism in other host-microbiota interactions.

## Materials and Methods

### C. elegans strains and maintenance

All worm strains were provided by the Caenorhabditis Genetics Center (CGC), which is funded by NIH Office of Research Infrastructure Programs (P40 OD010440). Strains included *wildtype* (N2 strain), *daf-2 (e1370)* and *daf-16 (mu86)*. *Daf-2 (e1370)* mutants were grown at 15 °C (Yan, 2017) and *daf-16 (mu86)* mutants were grown at 20 °C. We chose to grow *daf-2 (1370)* worms at 15 °C to limit the number of worms entering dauer phase; this allele of *daf-2* maintains wild type progeny and a long lifespan phenotype compared to N2 worms at 15 °C^61,62^. However, temperature dependent growth differences may still factor into comparisons. *N2* worms grown at 15 °C (WT-15) and 20 °C (WT-20) as control groups. All worms were grown on Nematode Growth Media (NGM) plates (0.25% Peptone, 51 mM NaCl, 25 mM [KPO4], 5 ug/ml cholesterol, 1 mM CaCl_2_, 1 mM MgCl_2_, 2% Agar). Plates were seeded with *E*. *coli* OP50, which was prepared by inoculating LB broth with OP50 and grown overnight, approximately 16 hours, at 37 °C^58^. All worms were synchronized using a sodium hypochlorite preparation^63^. All *C*. *elegans* were grown for three days from L1 synchronization and then transferred to NGM plates containing 50 μM 5-Fluoro-2’-deoxyuridine (FUdR, Alfa Aesar). FUdR inhibits progeny growth, which allows for the collection of animals of the appropriate age. After 24 hours, approximately 1000–1200 4-day old worms were washed six times with M9 buffer solution (3 g KH2PO4, 6 g Na2HPO4, 5 g NaCl, 1 ml 1 M MgSO4) and collected. The final wash solution showed bacterial RNA below detection, suggesting that most bacteria on the surface of the worm was washed away. For bacteria grown *in vitro*, OP50 was seeded on NGM + FUdR plates, allowed to dry at room temperature for approximately 24 hours, placed in 20 °C, and washed for RNA preparation after 3-days. Each group was prepared and collected in triplicate and subject to RNA extraction.

### Transcriptome library preparation and sequencing

Samples were organized into six groups for transcriptome sequencing and analysis, in which triplicate samples were processed for each group. Two of the six groups were *in vitro* experiments with *E*. *coli* OP50 grown on control NGM plates and *E*. *coli* OP50 grown on NGM plates supplemented with 50 μM FUdR. The remaining four groups consisted of transcriptome samples from *C*. *elegans* genotypes including: wildtype worms grown at 15 °C and 20 °C (WT-15 and WT-20, respectively), *daf-2*/Insulin/IGF-1 like receptor mutants grown at 15 °C (*daf-2*), and *daf-16*/FOXO transcription factor mutants grown at 20 °C (*daf-16*). For RNA extraction, worm samples were thawed on ice, crushed in M9 buffer using a 200 uL pipette tip to access gut contents of the worm, and centrifuged at 3000 rpm for 3 minutes. The centrifugation was repeated three times using the same supernatant. After the third centrifugation, the supernatant was removed and placed into a fresh microcentrifuge tube and used for the Powermicrobiome RNA isolation kit (Qiagen, CA) to extract RNA from the bacterial enrichment. Total RNA yielded from *in vivo* samples ranged from 2.2 ng/uL-5.6 ng/uL. Subsequently, ribosomal RNA was removed using the Ribo-Zero GOLD rRNA removal kit (Illumina, CA). The rRNA-subtracted samples underwent cDNA preparation and purification using Ambion Message AMP (Thermo Fisher, MA). cDNA concentrations were measured using the Qubit fluorometer (Thermo Fisher, MA). Equal amounts of (1 ng) of cDNA were added to Nextera XT library preparation kit (Illumina, CA) and were subject to dual index-barcoding. The quality of the transcriptome library was assessed by Agilent DNA 7500 Kit on the Agilent 2100 Bioanalyzer (Agilent, CA). Equimolar amounts of library were pooled and purified using QIAquick gel purification kit (Qiagen, CA). Purified libraries underwent sequencing on the Illumina HiSeq4000 following a SE100 rapid run.

### Quality assessment and filtering

The quality of raw data from sequencing was evaluated using the program FastQC and generated average Q scores across the read length of all sequence files. Trimmomatic (Version 0.36) was used to quality filter raw reads using a sliding window filtration to truncate reads at a 4-base average Q score of 20 or lower^64^. Reads trimmed below 80 basepairs were discarded. Filtered reads were run through KneadData (Version 0.5.4) to remove any potential human contaminant reads, *C*. *elegans* sequences, as well as ribosomal RNA reads. A customized bowtie2 index using a publicly available *C*. *elegans* genome assembly (accession PRJNA13758) was generated and mapped against to remove *C*. *elegans* sequences. Standard filtration parameters for KneadData rRNA filtration were implemented using the SILVA (release 128) rRNA database as a reference.

### Sequence assembly and alignment

Assembly was conducted using Trinity RNA-seq De novo Assembly tool following alignment of filtered reads to *E*. *coli* OP50 (accession PRJNA41499) genome^65^. Filtered reads were aligned using TopHat2^66^. Both the *E*. *coli* OP50 genome, and the *C*. *elegans* (accession PRJNA13758) genome were used as reference genomes to check the number of reads aligned to host DNA (*C*. *elegans*) versus bacteria (*E*. *coli*). The percentage of aligned reads for each sample were recorded for both genomes. A low genome N50 of the available *E*. *coli* OP50 genome resulted in the need for annotation using the HUMAnN2 pipeline.

### Functional gene annotation

HUMAnN2 was used to map reads to the *Uniref50* database to identify functional genes and pathways within each sample^67^. Functional genes that mapped to *E*. *coli* were used for downstream analyses and were also regrouped as KEGG orthology (KO) terms. *E*. *coli* annotations underwent CPM (counts per million) normalization in HUMAnN2 for LEfSe analysis, PLS-DA, iPath data mapping, and pathview plotting^68^. Metacyc functional pathway information was also generated from Uniref50 data mapping within HUMAnN2, which was CPM normalized prior to LEfSe analysis.

### Functional data analysis

Sample-to-sample distance was calculated considering CPM normalized *E*. *coli* KO annotations using the R package *PoiClaClu*^69^. Poisson distances between samples were visualized in a heatmap using the R package *Pheatmap* (Version 1.0.10). The PLS-DA analysis was then generated using mixOmics, and visualized with ggplot2 and vegan packages in Rstudio^70^. CPM normalized counts of *E*. *coli* KEGG annotations were again utilized as input for PLS-DA analysis to compare host-associate and pure culture samples. For the PLS-DA analysis, the model was trained using a ten-fold cross-validation. Linear discriminant analysis effect size (LEfSe) was used to identify over and under expressed *E*. *coli* functional genes (KOs) between FUdR inoculated *in vitro* samples and host-associated samples. Pairwise LEfSe analyses were conducted between *in vitro* replicates and each respective host-associated cohort (*daf-2*, *daf-16*, *WT/N2*). Lists of significantly differential (LDA > 0.25, P < 0.05) functional genes were collated and redundant enriched genes were filtered. The collated lists of functional genes enriched within host-associated samples and FUdR inoculated *in vitro* samples were uploaded to the online functional pathway mapping tool iPath 3 for visualization^71^.

LEfSe was used to compare enrichment of Metacyc functional pathways between WT-15 and *daf-2* cohorts, as well as WT-20 and *daf-16*. The parameters used include a linear discriminant analysis (LDA) score of 0.50, an alpha value of 0.05 for the factorial Kruskal-Wallis test among classes and an alpha value of 0.05 for the pairwise Wilcoxon test between subclasses.

Identified KEGG functional genes were mapped to the *E*. *coli* Biofilm Formation functional gene pathway using the tool Pathview (R version 3.2.1)^72^ l. Average CPM normalized KO counts within *daf-16* worms and respective WT-20 samples were log transformed and displayed in a single Pathview plot. Each box represents a gene in the biofilm formation pathway and the different color indicates level of expression. For Venn diagram comparisons, first, CPM normalized KOs for wild types and mutants were filtered to remove KOs that had a CPM value of zero across all three replicates. KOs that had a value > 0 in at least one of the three replicates were retained. Afterwards, an online tool, Venny (2.1.0), was used to compare KOs in *daf-2* and WT-15 and identify the number of unique and overlapping genes. The same was done for *daf-16* and WT-20.

## Supplementary information

Supplementary Figure 1

Supplemental Table 1

Supplemental Table 2

Supplemental Table 3

Supplemental Table 4

Supplemental Table 5

Supplemental Table 6

Supplemental Table 7

Supplemental Table 8

Supplemental Table 9

Supplemental Data 1

## Data Availability

All fastq and metadata information has been made available on the NCBI short read archive (*SUB4136940*).
