# Supplementary material for: Using Bacterial Transcriptomics to Investigate Targets of Host-Bacterial Interactions in Caenorhabditis elegans
Source: Sci Rep. 2019 Apr 3;9:5545. doi: 10.1038/s41598-019-41452-2 (PMC6447554; doi:10.1038/s41598-019-41452-2)
Supplement: Supplementary file 1 — Supplementary Figure 1 [file 41598_2019_41452_MOESM1_ESM.pdf]

Using Bacterial Transcriptomics to Investigate Targets of Host-Bacterial Interactions in *Caenorhabditis elegans*

Running Title: Host-Bacterial Interactions in *Caenorhabditis elegans*

Jason P. Chan[1]#*, Justin R. Wright[1]#, Hoi Tong Wong[1], Anastasia Ardasheva[1], Jamey Brumbaugh[1], Christopher McLimans[1], Regina Lamendella*[1]

[1]Department of Biology, Juniata College, Huntingdon, PA, USA
[#]These authors contributed equally to this work

Disclosures: The content is solely the responsibility of the authors and does not necessarily represent the official views of the National Institutes of Health.

Corresponding Authors:
Regina Lamendella, PhD
Juniata College
Biology Department
1700 Moore Street
Huntingdon, PA 16652 USA
Tel #: 814-641-3553
Email: lamendella@juniata.edu

Jason Chan, PhD
Juniata College
Biology Department
1700 Moore Street
Huntingdon, PA 16652 USA
Tel #: 814-641-3738
Email: chan@juniata.edu

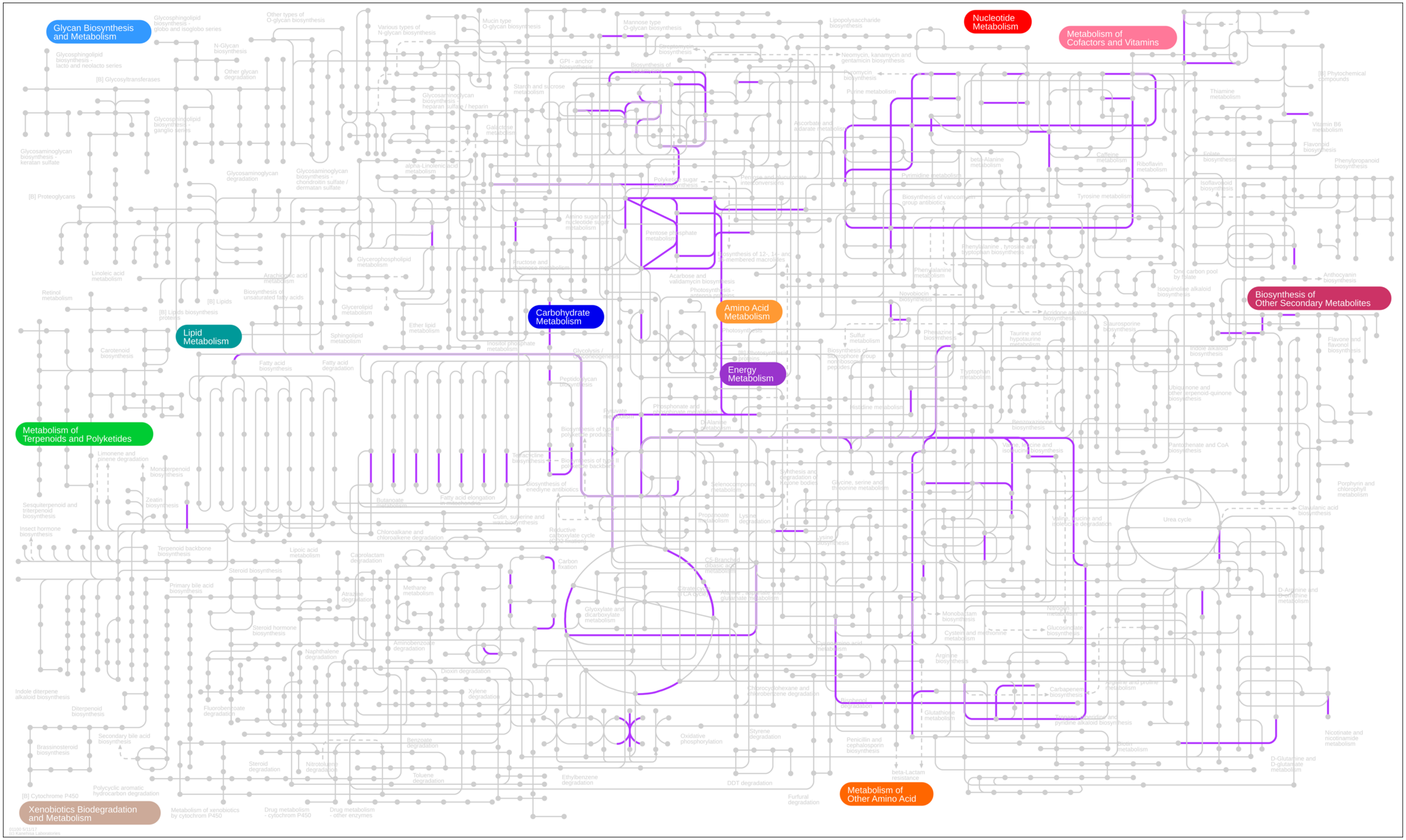

Supplemental Figure 1. iPath3 metabolic pathway mapping results of *core genes* identified across all 18 tested samples. *E. coli* core gene expression analysis revealed 192 total core KEGG orthologies[73-75]. 65 were successfully mapped to the iPath 3 Metabolic Pathway map and are highlighted in purple. Conserved gene expression of genes involved in the Pentose Phosphate Pathway including Transketolase A/B (tktA,tktB), transaldolase A/B (talA,talB) and 6-phosphofructokinase 1 (pfkA) can be observed.
